# Fine-scale monitoring of insecticide resistance in *Aedes aegypti* (Diptera: Culicidae) from Sri Lanka and modeling the phenotypic resistance using rational approximation

**DOI:** 10.1186/s13071-023-06100-9

**Published:** 2024-01-12

**Authors:** B. A. N. Mendis, V. Peiris, W. A. K. Harshani, H. S. D. Fernando, B. G. D. N. K. de Silva

**Affiliations:** 1 Center for Biotechnology, Department of Zoology, Faculty of Applied Sciences, University of Sri Jayewardenepura, Nugegoda, Sri Lanka; 2 Genetics and Molecular Biology Unit, Faculty of Applied Sciences, University of Sri Jayewardenepura, Nugegoda, Sri Lanka; 3 Deakin University, 221 Burwood Hwy, Burwood, VIC 3125 Australia; 4 Center for Optimization and Decision Science, Curtin University, Kent Street, Bentley, WA 6102 Australia

**Keywords:** *Aedes aegypti*, *kdr*, Insecticide resistance, Pyrethroids, Rational approximation, Sri Lanka

## Abstract

**Background:**

The unplanned and intensified use of insecticides to control mosquito-borne diseases has led to an upsurge of resistance to commonly used insecticides. *Aedes aegypti*, the main vector of dengue, chikungunya, and Zika virus, is primarily controlled through the application of adulticides (pyrethroid insecticides) and larvicides (temephos). Fine spatial-scale analysis of resistance may reveal important resistance-related patterns, and the application of mathematical models to determine the phenotypic resistance status lessens the cost and usage of resources, thus resulting in an enhanced and successful control program.

**Methods:**

The phenotypic resistance for permethrin, deltamethrin, and malathion was monitored in the *Ae. aegypti* populations using the World Health Organization (WHO) adult bioassay method. Mosquitoes' resistance to permethrin and deltamethrin was evaluated for the commonly occurring base substitutions in the voltage-gated sodium channel (*vgsc*) gene. Rational functions were used to determine the relationship between the *kdr* alleles and the phenotypic resistant percentage of *Ae. aegypti* in Sri Lanka.

**Results:**

The results of the bioassays revealed highly resistant *Ae. aegypti* populations for the two pyrethroid insecticides (permethrin and deltamethrin) tested. All populations were susceptible to 5% malathion insecticide. The study also revealed high frequencies of C1534 and G1016 in all the populations studied. The highest haplotype frequency was detected for the haplotype CC/VV, followed by FC/VV and CC/VG. Of the seven models obtained, this study suggests the prediction models using rational approximation considering the *C* allele frequencies and the total of *C*, *G*, and *P* allele frequencies and phenotypic resistance as the best fits for the area concerned.

**Conclusions:**

This is the first study to our knowledge to provide a model to predict phenotypic resistance using rational functions considering *kdr* alleles. The flexible nature of the rational functions has revealed the most suitable association among them. Thus, a general evaluation of *kdr* alleles prior to insecticide applications would unveil the phenotypic resistance percentage of the wild mosquito population. A site-specific strategy is recommended for monitoring resistance with a mathematical approach and management of insecticide applications for the vector population.

**Graphical Abstract:**

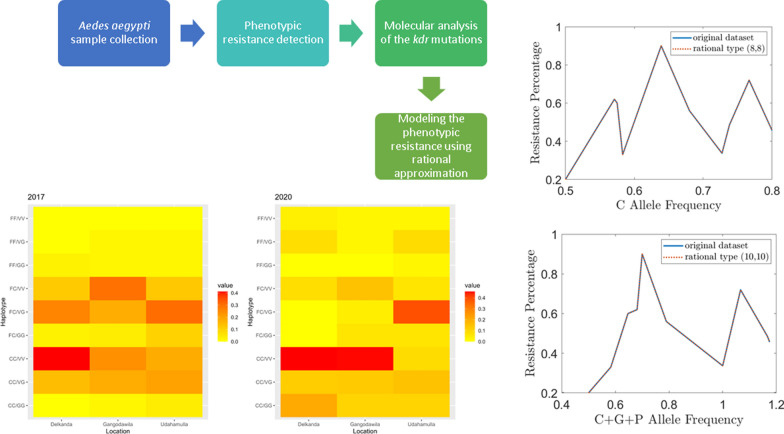

**Supplementary Information:**

The online version contains supplementary material available at 10.1186/s13071-023-06100-9.

## Background

Mosquitoes are considered to be one of the main vectors responsible for transmitting life-threatening vector-borne diseases to humans [1]. Control of these vector mosquitoes mainly relies on using insecticides. However, unplanned and widespread usage of insecticides has led to the rise and spread of resistance to currently used insecticides in major mosquito vectors. *Aedes aegypti* (Linnaeus) is the main vector of recent dengue outbreaks in tropical countries [2]. It is an aggressive, daytime-biting mosquito, highly anthropophilic, and thus adapted to breeding in a variety of artificial containers in urban and semi-urban environments [3].

Dengue fever (DF) and dengue hemorrhagic fever (DHF) epidemics have plagued Sri Lanka for over 2 decades. Since the middle of the 1960s, dengue infections have been endemic in Sri Lanka because of the *Aedes aegypti* and *Ae. albopictus* mosquitoes [4]. As of November 2023, the number of reported dengue cases for the year 2023 has risen to 75,000. Colombo district exhibits a significant number of incidences securing about 21% of the total cases. In 2021, the number of total cases recorded was 36,120, while in 2022 it surged to 76,467 [5]. Vector control is mainly done by source reduction of larval habitats and chemical control using adulticides and larvicides [4]. The pyrethroid group of insecticides is the most prevalent group of insecticides used by both private and government sectors against *Aedes* adult mosquitoes in most tropical countries, including Sri Lanka, because of their efficacy and low mammalian toxicity [5]. Abate^®^ (temephos) is used for larval breeding sites while PestGuard^®^ 161 has been the approved adulticide used since 2009 [5].

Target site or knockdown resistance (*kdr*) mutations in the sodium channel gene are the main mechanisms that compromise the susceptibility to pyrethroid insecticides [6]. These *kdr* mutations have been detected widely in mosquito vectors following the first discovery of the L1014F mutation in the housefly, *Musca domestica* [7]. With strong and continuous selection pressure, the mutant-resistant alleles will increase in frequency, although there is a fitness cost regarding the upsurge of resistant mutations in *Ae. aegypti* [8]. Multiple point mutations (synonymous and non-synonymous) of *vgsc*, namely V410L, S989P, I1011M/V, V1016G/I, I1532T, F1534S/L/C, D1763Y, G923V, L982W, S989P, and T1520I, have been detected in previous studies in pyrethroid-resistant *Ae. aegypti* mosquito populations worldwide [9, 10]. Nevertheless, only seven non-synonymous mutations have been functionally confirmed to have been associated with pyrethroid resistance, namely S989P, I1011M, V1016G, F1534C, V410L, L982W, and F1534L in electrophysiological studies [11, 12]. Co-occurrence of the *kdr* mutations has been common in many countries and has been shown to confer higher levels of resistance than singularly occurring mutations. Haplotypes containing V1016G/F1534C alleles have been reported in Thailand, Myanmar, Malaysia, and Indonesia [13–16]. In contrast, haplotypes containing V1016G/S989P alleles have been reported in Thailand, Myanmar, Indonesia, and Papua New Guinea [14, 17–19]. The triple haplotype allele combination F1534C/V1016G/ S989P has been recorded in Sri Lanka, Thailand, Myanmar, Saudi Arabia, and Indonesia [10, 14, 15, 17, 20–24].

Vector management programs require knowledge of the phenotypic and genotypic resistance status of the commonly used insecticides and the resistance mechanisms of the vector mosquitoes [25]. Previous research on *kdr* and insecticide resistance in field populations has primarily extrapolated to a larger geographic region. Therefore, sampling was done only in a few areas in the country, which offered a glimpse of the resistance scenario in the country or a bigger geographic area as a whole [26–28]. However, recent studies have suggested a strong variability in the insecticide selection pressure on a finer, more specific scale and time because of the extent and frequency of highly focused insecticide applications [29, 30]. Thus, insecticide resistance dynamics need to be established and understood on a finer spatial scale.

Phenotypic assessment of resistance is conducted via bioassay tests. This is the most popular and currently considered the most reliable way of assessing the resistance in field-collected mosquitoes [31]. However, phenotypic assessment requires many live mosquitoes of fixed age, and the population needs to be sampled evenly to avoid inbreeding bias. Thus, genotyping mosquitoes to extrapolate their resistance level would provide a convenient and simple way of assessing the resistance status of a particular mosquito population. Mathematical models are mostly used to build relationships among various aspects related to diseases. The application of mathematical models in epidemiology research commenced back in 1970 [32] and was mostly used to understand disease dynamics, evaluate control measures, and forecast disease outbreaks [33, 34]. In this study, classical rational function, the ratio of two polynomial functions where the denominator is not zero for inferring the relationship between the insecticide resistance and mutations present in the dengue vector, is considered. The rational functions are applied in many real-life applications, such as remote sensing and photogrammetric processing, because of their ability to correlate factors which change abruptly [28].

The present study was carried out to observe and understand the relationship between pyrethroid resistance phenotype and genotype frequencies in fine scale/Medical Officer of Health (MOH) divisions, where surveying dengue patients and control activities operate in the country. The aim of the present study is to develop a *kdr* genotypic model that can be used to extrapolate the phenotypic resistance status of an *Ae. aegypti* population. To the best of our knowledge, this is the first study of its kind.

## Methods

### Study site

**Fig. 1:**
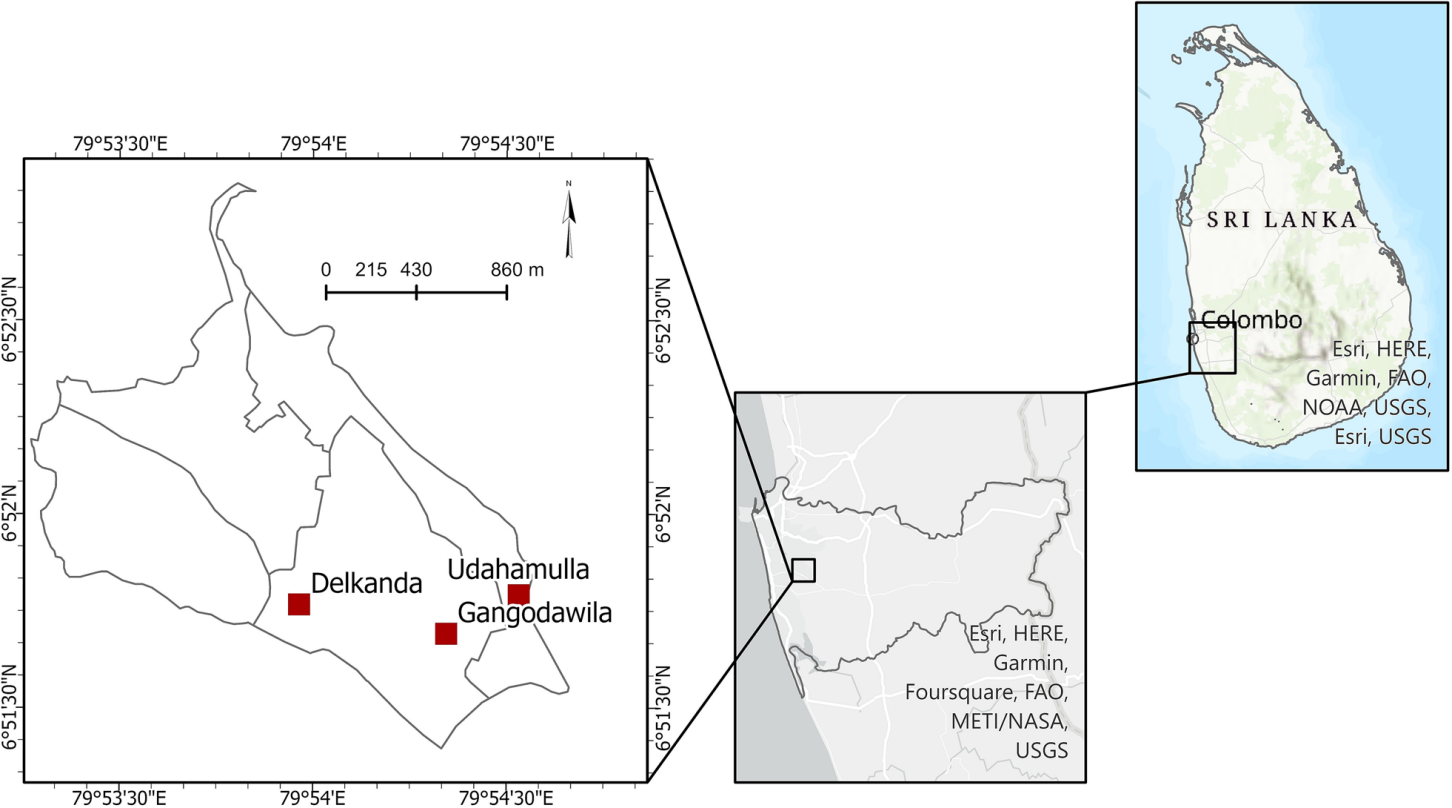
Sample collection sites for *Aedes aegypti* from Gangodawila PHI area of Colombo district, Sri Lanka

In Sri Lanka, each administrative district is divided into MOH divisions, and each MOH is divided into smaller Public Health Inspector (PHI) areas. The public health inspectors implement dengue control activities in the PHI areas, the smallest administrative division. For the present study, the Gangodawila PHI area of the Nugegoda MOH area in the Colombo district was selected as the study site (Fig. 1). The Gangodawila PHI area was divided into three main study areas, Delkanda, Gangodawila, and Udahamulla, according to the number of dengue incidences recorded during the year 2016. The study area comprised an area of about 2.86 km^2^ with a population of 21,754 inhabitants (population density of 7606 inhabitants per km^2^). The Gangodawila PHI area is considered a high-risk area for dengue in the Nugegoda MOH area. Therefore, control measures are led by peri-domestic insecticide spraying (pyrethroids) for adult mosquitoes and the application of larvicides (temephos) for larval stages of the vector. Although these measures, including regular vector surveillance programs, are implemented, the number of dengue patients has increased significantly over the years.

### Mosquito sampling and rearing

The mosquito eggs, larvae, and pupae samples were collected from each study area in 2017 and 2020. Sampling was carried out at the same sites during both years. Samples were collected using randomly placed ovitraps in the study sites, and each premise was additionally checked for the presence of *Ae. aegypti* breeding. In total, 100 ovitraps were placed per sampling site, maintaining 50 m distance between each site [35]. At each site, the ovitraps were placed both inside and outside of the randomly selected houses. Ovitraps were inspected after 5 days, and collected samples were transported to the insectary, Department of Zoology, University of Sri Jayewardenepura, and reared to adults at a temperature of 28 ± 2 °C and relative humidity of 70–80%. The larvae were fed with fish feed, and the emerged and the field-collected pupae were put in small plastic cups with labels indicating the location and date. Eggs and preimaginal stages collected from the ovitraps and entomological surveys in each study site were pooled to generate a single population for each site. From the emerged adults, *Ae. aegypti* was identified using standard taxonomic keys [36]. The emerged adults were fed with a 10% sucrose solution [37]. After mating between male and female mosquitoes, the female mosquitoes of F_0_ progeny were fed with human blood to induce egg laying using an artificial membrane feeder [37]. Oviposition cups with filter papers partially submerged in distilled water were placed as oviposition substrate in the cages. After oviposition, the cups were removed from the cages and the filter papers containing eggs were dried. The filter papers were then placed in zip lock bags [38]. Subsequently, F1 mosquitoes were used for adult bioassays [10].

### Adult susceptibility test for insecticide resistance

Adult insecticide susceptibility tests were carried out for each population of three study areas using the WHO standard bioassay kits. Three- to 5-day-old, non-blood-fed, sucrose-fed adult female *Ae. aegypti* mosquitoes from F1 progeny were used for all the adult bioassay experiments. About 125 mosquitoes were used for each adult bioassay, which was carried out at 25 ± 2 °C. An experiment included four replicates each consisting of 25 mosquitoes per tube and one control tube.

The adult susceptibility test procedure for the mosquitoes collected in 2017 was carried out using three WHO standard concentrations of insecticide-treated filter papers of 0.05% deltamethrin and 0.75% permethrin belonging to the pyrethroid (PY) group and 5% malathion belonging to the organophosphate (OP) group, which were purchased from WHO affiliated Universiti Sains Malaysia [39]. However, as WHO-recommended dosages for testing *Aedes* sp. mosquito susceptibility have changed, the experiments carried out in the year 2020 were done using the newly recommended dosages and their 5× concentration as deltamethrin 0.03% and 0.15%, permethrin 0.25% and 1.25%, and malathion 0.8% [40].

In the exposure tubes, mosquitoes were exposed for 1 h to insecticide-impregnated papers. They were subsequently placed in holding tubes and given a 10% w/v sucrose solution. After 24 h, the number of dead mosquitoes and those alive yet incapable of moving in holding tubes were counted as Susceptible (*S*), and the remaining adult mosquitoes who survived were counted as Resistant (*R*) mosquitoes [41].

### DNA extraction and kdr mutation genotyping

Genomic DNA was extracted from 25–30 pyrethroid-resistant *Ae. aegypti* mosquitoes from each area using a modified phenol/chloroform extraction method. Extracted DNA was stored in 100 µl Tris EDTA (TE) buffer. The presence of the *kdr* mutations occurring at 1534 and 1016 positions of the *vgsc* was assessed using allele-specific polymerase chain reaction (AS-PCR) for all the samples of 2017 and 2020 [42].

### DNA sequencing

To confirm the presence of V1016G and S989P *kdr* mutation in the 2017 samples, a fragment of Domain II subunit 6 in *vgsc* that contained the mutation was amplified and sequenced. Each reaction was performed in a 25 μl reaction volume with 1.5 mM MgCl_2_, 1 × PCR buffer, 0.5 μM of forward and reverse primers, 200 μM of dNTP mix, and 0.4 units of *Taq* DNA polymerase. The PCR thermocycle consisted of 95 °C for 2 min, followed by 35 cycles at 95 °C for 30 s, 63 °C for 30 s, and 72 °C for 30 s, followed by a final extension of 72 °C for 2 min [43]. Amplified products were visualized on 1.5% agarose gels (Tris–borate-EDTA; TBE) and were sent to Macrogen (Seoul, Korea) for sequencing.

For the F1534C mutation confirmation of the samples of the year 2017, a fragment of Domain III subunit 6 in *vgsc* that contained the mutation was amplified and sequenced. Each reaction was performed in a 25-μl reaction volume with 1.5 mM MgCl_2_, 1 × PCR buffer, 0.5 μM of forward and reverse primers, 200 μM dNTP mix, and 0.4 units of *Taq* DNA polymerase. The PCR thermocycle consisted of 95 °C for 2 min, followed by 35 cycles at 95 °C for 30 s, 63 °C for 30 s, and 72 °C for 30 s, followed by a final extension of 72 °C for 2 min [43]. Amplified products were visualized on 1.5% agarose gels (TBE) and were sent to Macrogen (Seoul, Korea) for sequencing.

### Statistical analysis

Susceptibility was categorized according to the resulting mortality percentage using WHO guidelines: 98–100% mortality denotes susceptibility; 90–97% mortality denotes possible resistance; < 90% mortality denotes resistance [44] for diagnostic doses of insecticides. Furthermore, the intensity of insecticide resistance was categorized according to the resulting mortality percentage using WHO guidelines: ≥ 98% mortality denotes low-intensity resistance, and < 98% mortality denotes moderate to high-intensity resistance for 5 × doses of insecticides [40].

To visualize the frequency of different haplotypes reported at F1534C and V1016G, in the three areas in 2017 and 2020, heat maps were prepared using version 4.3.1 of the R statistical software [45].

Regression plots were prepared using ggplot2 in R software to infer the linear relationship between the resistance allele frequencies and insecticide resistance [45].

### Preparation of the mathematical model

This section of experiments aimed to investigate the relationship between alleles and their linear combinations with the phenotypic resistance percentage. In particular, three different allele frequencies, *C*, *G*, and *P*, were considered. In addition, summations of two or more allele frequencies were assessed, and their relationship with the resistance percentage was investigated. Mathematical models were developed to quantify the relationship between each set of variables.

The approach of finding the corresponding mathematical models was different from what is available in the literature. Rational approximation techniques, which are simple and robust, were used to build the models [46]. Rational functions are very efficient and powerful, so that the models generated by rational approximations are very accurate, especially in such cases where the number of data points in the dataset is limited in size. This type of small dataset is very common in practice, particularly when each observation results from a very expensive experiment. At the same time, the accuracy and reliability of such small data sets are very high since every experiment is carefully designed and analyzed. On the other hand, it is also possible that some or all of these accurate data points of a small set belong to an under-represented group. The so-called regression curves are usually, incapable of modeling such limited data sets since they tend to average the errors and automatically lead the model to ignore the under-represented groups. Based on these observations, rational approximations in uniform norm are the best method of approximation in this case.

Peiris et al. [46] utilized a method called ‘the bisection method’ to find the best rational approximations uniformly. The error term is usually known as the ‘uniform error.’ The aim is to build models such that the maximum absolute errors between the original values and the values computed by the model are minimized.

The resistance data from the Gangodawila PHI in 2017 (133 mosquito samples) and a similar study carried out in Colombo, Gampaha, and Galle by Fernando et al. (2018) (281 mosquito samples) was used in the evaluation [10].

Since the dataset is small, we utilize the whole dataset for the experiments.

In experiments, the following seven models were considered.

- Model 1: Relationship between *C* allele frequency and the resistance percentage
- Model 2: Relationship between *G* allele frequency and the resistance percentage.
- Model 3: Relationship between *P* allele frequency and the resistance percentage.
- Model 4: Relationship between *C* allele frequency + *G* allele frequency and the resistance percentage.
- Model 5: Relationship between *C* allele frequency + *P* allele frequency and the resistance percentage
- Model 6: Relationship between *G* allele frequency + *P* allele frequency and the resistance percentage.
- Model 7: Relationship between *C* allele frequency + *G* allele frequency + *P* allele frequency and the resistance percentage.

Trial and error were necessary to determine the degree of the rational function. To do that, for all the models, we started with a rational function of degree (10, 10) and checked whether the approximation produced by the bisection method was suitable. If not, the degree of the rational function was decreased until a suitable model was created. Note that the computational codes are implemented in MATLAB, version R2022b.

## Results

### Adult susceptibility test for insecticide resistance

**Fig. 2:**
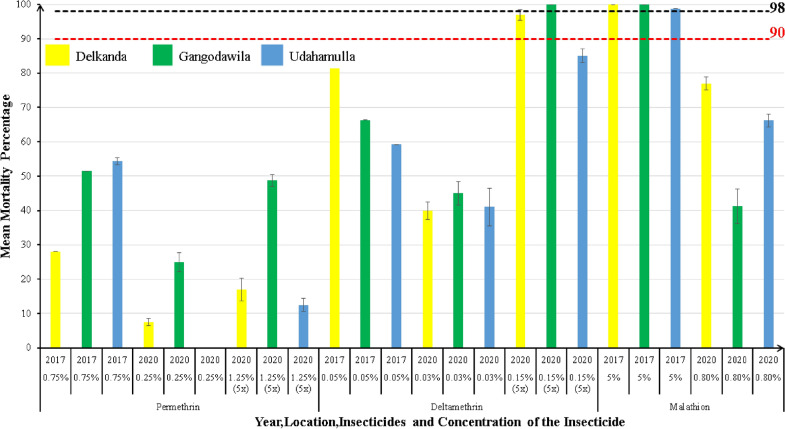
Susceptibility test results of adult bioassay in 2017 and 2020 at the Gangodawila PHI area. Permethrin, deltamethrin, and malathion were used to analyze samples in all three study sites

The highest mortality percentage for permethrin (permethrin 0.75%) was recorded from Udahamulla (54.35%) in 2017, whereas it was recorded from Gangodawila for both 0.25% and 1.25% concentrations (25% and 49%, respectively) in 2020 (Fig. 2). The highest mortality percentage for deltamethrin (deltamethrin 0.05%) was recorded from Delkanda (84.68%) in 2017. In contrast, it was recorded from Gangodawila for both 0.03% and 0.15% concentrations (45% and 100% respectively) in 2020. The highest mortality percentage for malathion (malathion 5%) was recorded from Delkanda and Gangodawila (100%) in 2017, whereas it was recorded from Delkanda (77%) for 0.8% concentration in 2020. The detailed mortality percentages for each locality are available in the Additional file 1: Table S1.

### kdr mutation genotyping

High frequencies of *C* and *G* allele were detected in all the populations studied (Table 1). The highest *C* allele frequency (0.9167) was recorded from the Gangodawila-resistant population in 2020, whereas the highest *G* allele frequency (0.5536) was from the Udahamulla permethrin-resistant population in 2020.

**Table 1:** Frequency of *kdr* alleles in permethrin and deltamethrin resistant *Aedes aegypti* mosquitoes

Site	Year	Pyrethroid phenotype	F1534C genotype					V1016G genotype				
			*n*	*F*/*F*	*F*/*C*	*C*/*C*	*C* allele frequency	*n*	*V*/*V*	*V*/*G*	*G*/*G*	*G* allele frequency
Gangodawila	2017	Permethrin resistant	25	2	11	12	0.7000	25	10	12	3	0.3600
	2020	Permethrin resistant	29	2	10	17	0.7586	27	20	5	2	0.1667
	2017	Deltamethrin resistant	25	0	15	10	0.7000	25	17	7	1	0.1800
	2020	Deltamethrin resistant	24	0	4	20	0.9167	21	10	5	6	0.4048
Udahamulla	2017	Permethrin resistant	27	1	10	16	0.7778	27	9	17	1	0.3519
	2020	Permethrin resistant	24	3	9	12	0.6875	28	5	15	8	0.5536
	2017	Deltamethrin resistant	32	3	23	6	0.5469	23	6	9	8	0.5435
	2020	Deltamethrin resistant	28	5	16	7	0.5357	25	4	17	4	0.5000
Delkanda	2017	Permethrin resistant	25	1	8	16	0.8000	24	15	7	2	0.2292
	2020	Permethrin resistant	21	3	1	17	0.8333	16	6	6	4	0.4375
	2017	Deltamethrin resistant	17	1	9	7	0.6765	11	3	8	0	0.3636
	2020	Deltamethrin resistant	24	2	6	16	0.7917	13	8	3	2	0.2692

The three genotypes of F1534C were the wild-type homozygous F/F, heterozygous F/C, and homozygous mutant C/C, while those of V1016G were wild-type homozygous V/V, heterozygous V/G, and homozygous mutant G/G. (F = phenylalanine; C = cysteine; V = valine; G = guanine).

**Fig. 3:**
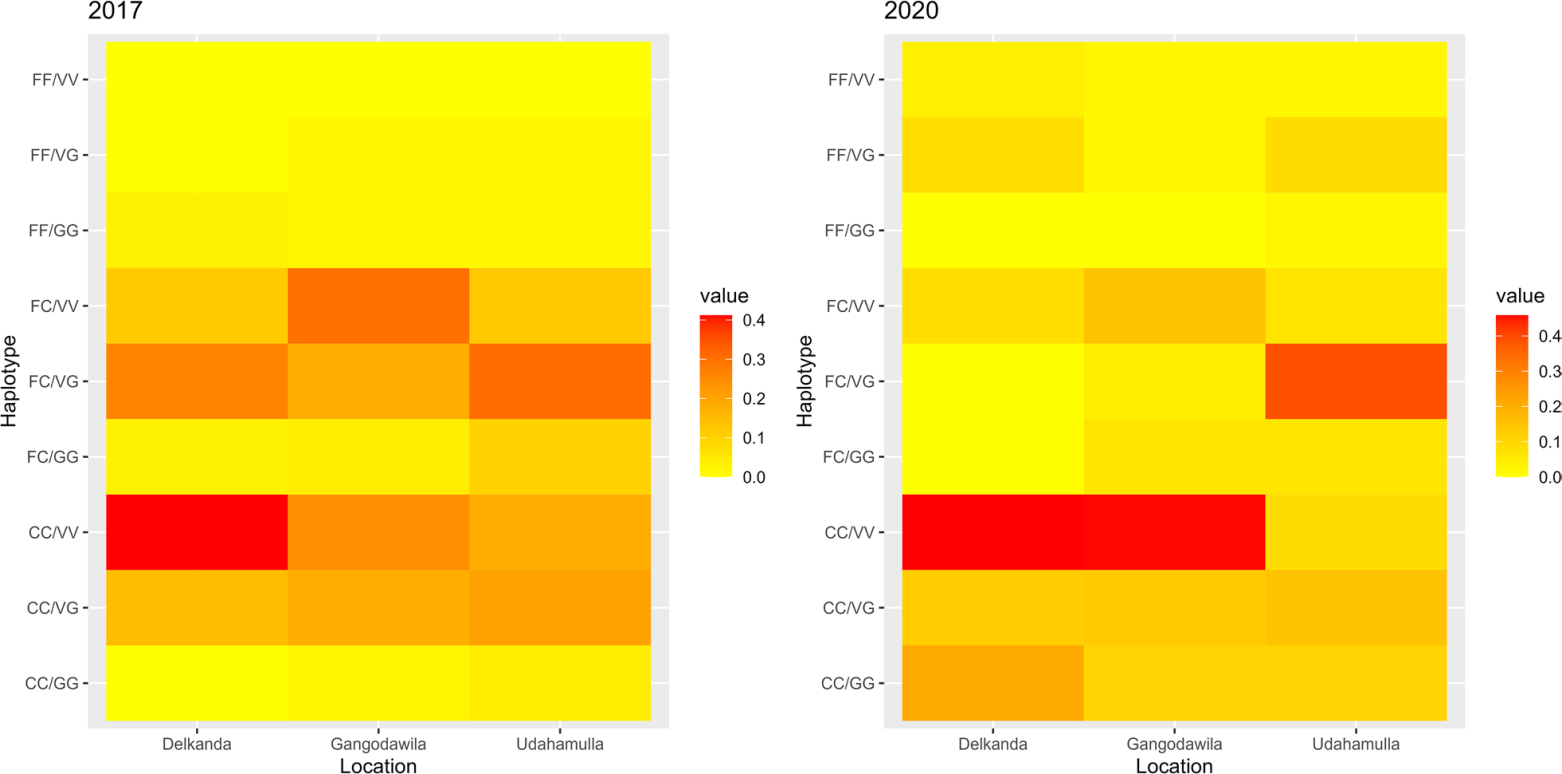
Heat maps representing the haplotype frequencies in the years 2017 and 2020 of the three study sites, Delkanda, Gangodawila, and Udahamulla

The haplotype frequencies per year in their respective geographic region are displayed in Fig. 3. Haplotype CC/VV was the highest recorded haplotype frequency in 2017 in Delkanda, while that of 2020 was CC/VV in Delkanda and Gangodawila areas. There is a visible increase in the double mutant homozygotes (CC/GG) in all three areas from 2017 to 2020. There is an apparent decline in the heterozygous haplotype FC/VG from 2017 to 2020. The detailed haplotype count and frequencies for each locality are available in the Additional file 2: Table S2.

When considering the 2017 data, regarding all three mutations, CC/VV/SS was the most abundant recorded from the Delkanda area (Additional file 3: Table S3). It was also the most abundant haplotype in all three areas. FF/GG/SS, CC/VV/SP, and CC/GG/SS were not recorded from any of the three study sites.

### Results of the mathematical model

**Fig. 4:**
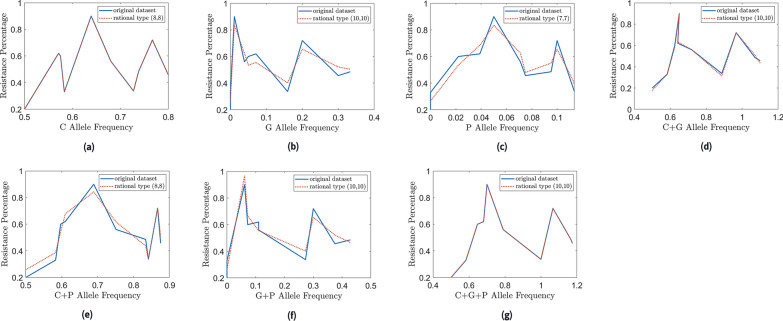
Mathematical models created for the original dataset. Correlation between the resistance and resistant alleles in *Aedes aegypti* mosquitoes in the year 2017 using the rational approximation technique

In this section, the results of the mathematical models are discussed in detail. Figure 4 includes the resulting plots of the mathematical models generated by the rational approximation against the original data set. Figure 4a, d and g clearly shows that the approximations coincide with the original dataset; therefore, the corresponding models are considered accurate.

#### Model 1: Relationship between *C* allele frequency and the resistance percentage

**Fig. 5:**
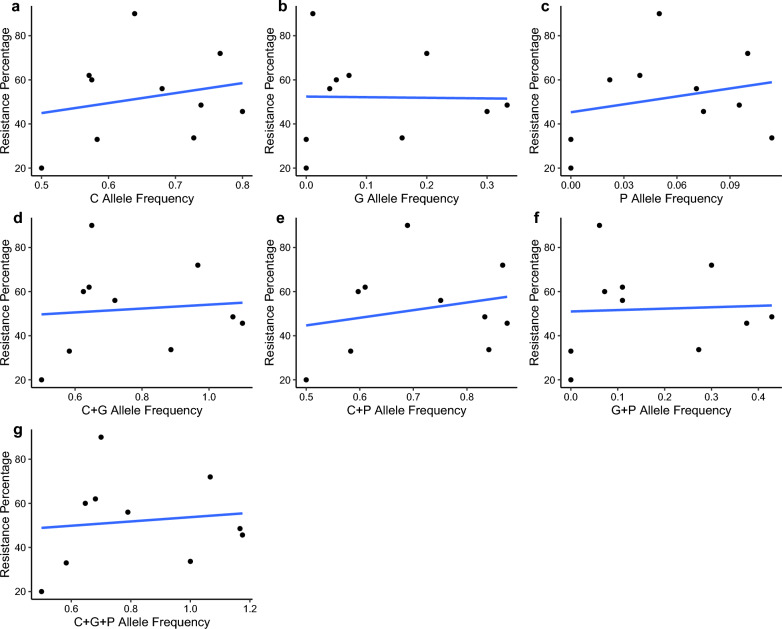
Linear regression lines. The plots reveal the association between the resistance and resistant alleles in *Aedes aegypti* mosquitoes in the year 2017 according to linear regression

Figure 4 a shows that the approximation (also known as the model) perfectly coincides with the original dataset. The error is 4.44788 × 10^–4^, which is very small compared to the error (21.25) computed by the corresponding linear regression line (Fig. 5a).

The model depicted by this curve is as follows:

1 $${\text{Resistance}} = \frac{{\left( {a_{0} + a_{1} C + a_{2} C^{2} + a_{3} C^{4} + a_{4} C^{6} + a_{5} C^{7} + a_{6} C^{8} } \right)}}{{\left( {1 + b_{1} C + b_{2} C^{2 } + b_{3} C^{3 } + b_{4} C^{5 } + b_{5} C^{7 } + b_{6} C^{8 } } \right)}}$$

The values of the coefficients of Model 1 are stated below (Table 2).

**Table 2:** The values of the coefficients of Model 1

Basis function	Coefficient	Value of the coefficient
1	${a}_{0}$	− 923296331.8627
$C$	${a}_{1}$	6380109874.0697
${C}^{2}$	${a}_{2}$	− 13473410689.6903
${C}^{4}$	${a}_{3}$	− 28532687959.5758
${C}^{6}$	${a}_{4}$	− 85005552114.6877
${C}^{7}$	${a}_{5}$	101202429859.4744
${C}^{8}$	${a}_{6}$	− 36864215471.3718
$C$	${b}_{1}$	977656565.50054
${C}^{2}$	${b}_{2}$	− 5549465100.10692
${C}^{3}$	${b}_{3}$	9462195446.17072
${C}^{5}$	${b}_{4}$	− 11956488868.64405
${C}^{7}$	${b}_{5}$	16094216688.79076
${C}^{8}$	${b}_{6}$	− 9133869507.80082

*C-C* allele frequency

#### Model 2: Relationship between *G* allele frequency and the resistance percentage.

Figure 4b shows the approximation model of resistance against *G* allele frequency. The error is 0.06502, while that of the linear regression is 21.78 (Fig. 5b). The model depicted by this curve is stated below:

2 $${\text{Resistance}} = \frac{{\left( {a_{0} + a_{1} G + a_{2} G^{2} + a_{3} G^{4} + a_{4} G^{5} + a_{5} G^{10} } \right)}}{{\left( {1 + b_{1} G^{3 } + b_{2} G^{5 } + b_{3} G^{7 } + b_{4} G^{10 } } \right)}}$$

The values of the *G* allele frequencies and coefficients of Model 2 are shown below (Table 3).

**Table 3:** The values of the coefficients of Model 2

Basis function	Coefficient	Value of the coefficient
1	${a}_{0}$	0.265
$G$	${a}_{1}$	72.623546
${G}^{2}$	${a}_{2}$	− 1753.282611
${G}^{4}$	${a}_{3}$	587661.614865
${G}^{5}$	${a}_{4}$	− 3658541.295479
${G}^{10}$	${a}_{5}$	2791813478.236963
${G}^{3}$	${b}_{1}$	22210.172599
${G}^{5}$	${b}_{2}$	317108.798456
${G}^{7}$	${b}_{3}$	− 73562577.313898
${G}^{10}$	${b}_{4}$	6460398372.242500

*G*-*G* allele frequency

#### Model 3: Relationship between *P* allele frequency and the resistance percentage

Figure 4c contains the approximation model of resistance against *P* allele frequency. The error is 0.065, which is very close to the error found in model 2. Nevertheless, the error in linear regression is 21.15 (Fig. 5c). The model depicted by this curve is stated below:

3 $${\text{Resistance}} = \frac{{\left( {a_{0} + a_{1} P + a_{2} P^{2} + a_{3} P^{3} + a_{4} P^{5} + a_{5} P^{7} } \right)}}{{\left( {1 + b_{1} P^{3 } + b_{2} P^{4 } + b_{3} P^{6 } + b_{4} P^{7 } } \right)}}$$

The values of the *P* allele frequencies and coefficients of Model 3 are stated below (Table 4).

**Table 4:** The values of the coefficients of Model 3

Basis function	Coefficient	Value of the coefficient
1	${a}_{0}$	0.265000000
$P$	${a}_{1}$	21.770418667
${P}^{2}$	${a}_{2}$	− 689.751733001
${P}^{3}$	${a}_{3}$	4574.275487255
${P}^{5}$	${a}_{4}$	100363.819053150
${P}^{7}$	${a}_{5}$	− 9832564.821468228
${P}^{3}$	${b}_{1}$	− 20761.7526681
${P}^{4}$	${b}_{2}$	351809.4233696
${P}^{6}$	${b}_{3}$	− 27622486.3631150
${P}^{7}$	${b}_{4}$	124158795.6834685

*P*-*P* allele frequency

#### Model 4: Relationship between *C* allele frequency + *G* allele frequency and the resistance percentage

Figure 4d contains the approximation model of resistance against *C* allele frequency + *G* allele frequency. The approximation is much closer to the original function than model 2 and model 3. The error is 0.02291. The linear regression model exhibited an error of 21.69 (Fig. 5d).

The model depicted by this curve is as follows:

4 $${\text{Resistance}} = \frac{{\left( {a_{0} + a_{1} \left( {C + G} \right)^{2} + a_{2} \left( {C + G} \right)^{4} + a_{3} \left( {C + G} \right)^{5} + a_{4} \left( {C + G} \right)^{7} + a_{5} \left( {C + G} \right)^{9} + a_{6} \left( {C + G} \right)^{10} } \right)}}{{\left( {1 + b_{1} \left( {C + G} \right)^{ } + b_{2} \left( {C + G} \right)^{2 } + b_{3} \left( {C + G} \right)^{4 } + b_{4} \left( {C + G} \right)^{5 } + b_{5} \left( {C + G} \right)^{7 } + b_{6} \left( {C + G} \right)^{9} + b_{7} \left( {C + G} \right)^{10 } } \right)}}$$

The values of the coefficients of Model 4 are stated below (Table 5).

**Table 5:** The values of the coefficients of Model 4

Basis function	Coefficient	Value of the coefficient
1	${a}_{0}$	347231.89871471
${(C+G)}^{2}$	${a}_{1}$	− 5451048.06473052
${(C+G)}^{4}$	${a}_{2}$	51173428.72019885
${(C+G)}^{5}$	${a}_{3}$	− 85704569.96654052
${(C+G)}^{7}$	${a}_{4}$	76784470.23534042
${(C+G)}^{9}$	${a}_{5}$	− 68790001.27416702
${(C+G)}^{10}$	${a}_{6}$	31638575.86718503
$\left(C+G\right)$	${b}_{1}$	7890147.9804573
${(C+G)}^{2}$	${b}_{2}$	− 34553409.8652851
${(C+G)}^{4}$	${b}_{3}$	210801390.1094063
${(C+G)}^{5}$	${b}_{4}$	− 327273010.8525403
${(C+G)}^{7}$	${b}_{5}$	268191635.6432237
${(C+G)}^{9}$	${b}_{6}$	− 227546166.070013
${(C+G)}^{10}$	${b}_{7}$	102477501.8803313

*C* + *G* = Total of *C* and *G* allele frequencies

#### Model 5: Relationship between *C* allele frequency + *P* allele frequency and the resistance percentage

Figure 4e contains the approximation model of resistance against *C* allele frequency + *P* allele frequency. The error is 0.05706, while that of the linear regression is 21.19 (Fig. 5e). The model depicted by this curve is shown below:

5 $${\text{Resistance}} = \frac{{\left( {a_{0} + a_{1} \left( {C + P} \right) + a_{2} \left( {C + P} \right)^{2} + a_{3} \left( {C + P} \right)^{4} + a_{4} \left( {C + P} \right)^{6} + a_{5} \left( {C + P} \right)^{7} + a_{6} \left( {C + P} \right)^{8} } \right)}}{{\left( {1 + b_{1} \left( {C + P} \right)^{ } + b_{2} \left( {C + P} \right)^{3} + b_{3} \left( {C + P} \right)^{4 } + b_{4} \left( {C + P} \right)^{6 } + b_{5} \left( {C + P} \right)^{8} } \right)}}$$

The values of the *C* + *P* allele frequencies and coefficients of Model 5 are stated below (Table 6).

**Table 6:** The values of the coefficients of Model 5

Basis function	Coefficient	Value of the coefficient
1	${a}_{0}$	2268560.18995798
$\left(C+P\right)$	${a}_{1}$	− 13665908.18768946
${(C+P)}^{2}$	${a}_{2}$	25073847.09740878
${(C+P)}^{4}$	${a}_{3}$	− 39784443.81799661
${(C+P)}^{6}$	${a}_{4}$	88661124.33358555
${(C+P)}^{7}$	${a}_{5}$	− 91510924.12885486
${(C+P)}^{8}$	${a}_{6}$	28981251.69289512
$\left(C+P\right)$	${b}_{1}$	28981251.69289512
${(C+P)}^{3}$	${b}_{2}$	164958.059405934
${(C+P)}^{4}$	${b}_{3}$	− 2274557.572140106
${(C+P)}^{6}$	${b}_{4}$	4000282.615553982
${(C+P)}^{8}$	${b}_{5}$	− 3111181.159339364

*C* + *P* = Total of *C* and *P* allele frequencies

#### Model 6: Relationship between *G* allele frequency + *P* allele frequency and the resistance percentage

Figure 4f contains the approximation model of resistance against *G* allele frequency + *P* allele frequency. The error is 0.065. The error obtained from the linear regression is 21.76 (Fig. 5f).

The model depicted by this curve is shown below:

6 $${\text{Resistance}} = \frac{{(a_{0} + a_{1} \left( {G + P} \right) + a_{2} \left( {G + P} \right)^{4} + a_{3} \left( {G + P} \right)^{5} + a_{4} \left( {G + P} \right)^{10} )}}{{\left( {1 + b_{1} \left( {G + P} \right)^{ } + b_{2} \left( {G + P} \right)^{3 } + b_{3} \left( {G + P} \right)^{7 } + b_{4} \left( {G + P} \right)^{10} } \right)}}$$

The values of the *G* + *P* allele frequencies and coefficients of Model 6 can be found below (Table 7).

**Table 7:** The values of the coefficients of Model 6

Basis function	Coefficient	Value of the coefficient
1	${a}_{0}$	0.265000000
$\left(G+P\right)$	${a}_{1}$	− 7.610292612
${(G+P)}^{4}$	${a}_{2}$	28033.919141146
${(G+P)}^{5}$	${a}_{3}$	− 109436.419251507
${(G+P)}^{10}$	${a}_{4}$	6933109.075299110
$\left(G+P\right)$	${b}_{1}$	− 30.87308061
${(G+P)}^{3}$	${b}_{2}$	4346.23483591
${(G+P)}^{7}$	${b}_{3}$	− 1035659.71583039
${(G+P)}^{10}$	${b}_{4}$	19900821.93857696

*G* + *P* = Total *G* and *P* allele frequencies

#### Model 7: Relationship between *C* allele frequency + *G* allele frequency + *P* allele frequency and the resistance percentage

Figure 4g contains the approximation model of resistance against *C* allele frequency + *G* allele frequency + *P* allele frequency. The error is 6.85122 × 10^–4^, which is very low compared to the error 21.63 obtained from the linear regression (Fig. 5g).

The model depicted by this curve is as follows:

7 $${\text{Resistance}} = \frac{{\left( {a_{0} + a_{1} \left( {C + G + P} \right) + a_{2} \left( {C + G + P} \right)^{3} + a_{3} \left( {C + G + P} \right)^{5} + a_{4} \left( {C + G + P} \right)^{6} + a_{5} \left( {C + G + P} \right)^{8} + a_{6} \left( {C + G + P} \right)^{10} } \right)}}{{\left( {1 + b_{1} \left( {C + G + P} \right)^{ } + b_{2} \left( {C + G + P} \right)^{2 } + b_{3} \left( {C + G + P} \right)^{3} + b_{4} \left( {C + G + P} \right)^{5 } + b_{5} \left( {C + G + P} \right)^{6 } + b_{6} \left( {C + G + P} \right)^{8} + b_{7} \left( {C + G + P} \right)^{10 } } \right)}}$$

The values of the *C* + *G* + *P* allele frequencies and coefficients of Model 7 are stated below (Table 8).

**Table 8:** The values of the coefficients of Model 7

Basis function	Coefficient	Value of the coefficient
1	${a}_{0}$	716166607.22713
$\left(C+G+P\right)$	${a}_{1}$	− 2830518552.86041
${(C+G+P)}^{3}$	${a}_{2}$	10388466329.42499
${(C+G+P)}^{5}$	${a}_{3}$	− 37716698071.84403
${(C+G+P)}^{6}$	${a}_{4}$	40370145440.69236
${(C+G+P)}^{8}$	${a}_{5}$	− 13600454700.55536
${(C+G+P)}^{10}$	${a}_{6}$	02678494991.40093
$\left(C+G+P\right)$	${b}_{1}$	8017117875.1734
${(C+G+P)}^{2}$	${b}_{2}$	− 47249603859.7930
${(C+G+P)}^{3}$	${b}_{3}$	86218011175.7724
${(C+G+P)}^{5}$	${b}_{4}$	− 171072974438.1670
${(C+G+P)}^{6}$	${b}_{5}$	163788016008.2528
${(C+G+P)}^{8}$	${b}_{6}$	− 48590946801.6674
${(C+G+P)}^{10}$	${b}_{7}$	8907013521.3133

*C* + *G* + *P* = Total of *C*, *G*, and *P* allele frequencies

In Table 9, attributes of each model together with the corresponding uniform error terms are summarized for comparison.

**Table 9:** Summary of the models

Model	Independent variables	Uniform error term
1	*C* allele frequency	4.44788 × 10^–4^
2	*G* allele frequency	0.06502
3	*P* allele frequency	0.065
4	*C* allele frequency + *G* allele frequency	0.02291
5	*C* allele frequency + *P* allele frequency	0.05706
6	*G* allele frequency + *P* allele frequency	0.065
7	*C* allele frequency + *G* allele frequency + *P* allele frequency	6.85122 × 10^–4^

## Discussion

The current study was conducted at a fine geographical scale, and the results indicate variability in phenotypic resistance and resistant genotypes among the study sites. Although previous studies of *kdr* and phenotypic resistance in the field populations were conducted in a few locations in the whole country, and the results were extrapolated to the entire geographic area [10, 47–51], new studies have indicated the need to focus on a finer geographical scale in studying resistance development [29, 52, 53]. Grossman et al. [30] previously discovered this phenomenon with significant heterogeneity in the frequency of *kdr* haplotypes between city blocks of a dengue endemic town in Yucatan, where insecticide applications highly varied in space and time. Verhaeghen et al. [54] have reported seasonal and spatial fluctuations in *kdr* resistance genotypes. Similarly, a significant difference has been shown in phenotypic and genotypic resistance at a fine geographic scale in *Ae. aegypti* populations in Mexico [29]. Thus, it can be suggested that sampling and analysis of resistance on a finer geographic scale would reveal accurate and finer patterns of resistance. This would provide a broader picture of resistance patterns and evolutionary dynamics and help in determining appropriate control strategies.

All the adult bioassays of both years showed mortality percentages < 90% (except malathion mortality in 2017), indicating a low susceptibility/high resistance for diagnostic doses of commonly used insecticides in 2017 and 2020. A comparison between mortality percentages recorded for permethrin in both years indicated an increment of resistance after 3 years. Although the deltamethrin concentration used in 2017 (0.05%) was slightly higher than the concentration used in 2020 (0.03%), the mortalities recorded for deltamethrin were significantly less than in 2017, indicating resistance development. The frequently used adulticide in the area is a pyrethroid (Pesguard^®^), which may result in resistant development in *Aedes* populations. The concentration of malathion used in 2017 was 8%, and all the recorded mortalities were > 98%. In 2020, the recorded mortalities for malathion were significantly less than in 2017 (31–77% range), which could be because of the lower concentration (0.8%) used in 2020. This confirms the presence and upsurge of resistance to PY insecticides in the populations in the experiments. Hence, this suggests that the above-mentioned PY insecticides have subdued efficacy in controlling *Ae. aegypti*. However, when comparing the three insecticides, malathion showed a high mortality rate. The present study revealed that malathion has high effectiveness in controlling *Ae. aegypti*. Using insecticides on a rotational basis may reduce the resistance development of the dengue vectors [55].

Both F1534C mutation and V1016G mutation have been associated with resistance to pyrethroids in mosquito populations. C1534 allele has been strongly correlated with resistance to permethrin and dichlorodiphenyltrichloroethane (DDT), and recent literature has suggested the combined effect of the C1534 and G1016 alleles in resistance to pyrethroids [13]. In the populations studied, an increase in the C1534 and G1016 alleles is visible between the years. Though not significant, genotypic differentiation between the study sites indicates a difference in the resistance profiles of *Ae. aegypti* on a fine geographical scale. This scenario is particularly interesting in light of the population genetics study conducted in Sri Lanka, indicating a high level of gene flow in Colombo via passive transportation of mosquitoes and preimaginal stages [22]. This suggests focal insecticide application and household insecticides play an important role in maintaining insecticide pressure [56, 57].

Of seven models in the set of experiments, model 1 (*C* allele frequency vs resistance) is the best-fitted model with the lowest error term. Model 7 can also be concluded as an accurate model because of the nature of the error term. Model 4, whose independent variable is the total of *C* and *G* allele frequency, can also be predicted as an accurate model compared to the rest of the models. However, model 1 and model 7 would generate significantly better results than model 4. It is also noticeable that the recommended models strongly support the behaviors predicted by theory. In *Ae. aegypti*, V1016G and F1534C are the two main mutations proven to reduce the sensitivity of the *vgsc* to pyrethroid insecticides in both individual and combined capacity. Although S989P has not been proven to confer resistance directly, it has been suggested that the occurrence of all three mutations may reduce the susceptibility in higher degrees. Thus, it is interesting to note that the three models that can be concluded accurately predict the allele frequency between *C*, *G*, and *P* alleles and phenotypic resistance.

Rational approximations resulting in this study using the bisection method showed a close relationship fitting well with its dynamic nature. The reason for the well-fitting nature is the flexibility of rational approximation in the presence of small data sets. Generating mathematical models for small datasets is not an easy task. Even though regression models are widely used in practice, they may not be flexible enough to capture the features of a small dataset. In that sense, rational approximation is a better alternative.

The above results can be further improved by feeding larger datasets for the experiments. It is important to emphasize the fact that rational approximations have not been used in previous studies where the relationship between resistance and allele frequency has been investigated. This limits the ability to benchmark the present results to those of other research in this area. At the same time, the experiments are designed to compute the error terms in a uniform sense. Therefore, it is also impossible to compare the results with the regression curves as they use least squares errors.

In this study, only classical rational functions (ratio of two polynomials where the basis functions are monomials) were used when computing rational approximations. One can also change the format of the rational functions to generalized rational functions (ratio of two linear functions where the basis functions are not restricted to monomials) and carry out the same or different set of experiments. However, there is no rule of thumb to select suitable basis functions, and it remains an open problem in the field of approximation. Therefore, this research direction is left for future studies.

The current study results suggest the need to focus on the finer geographic scale when controlling the vector mosquitoes, which requires continuous monitoring of the insecticide resistance. The customary methods are expensive [58], and when the current insecticides need to be replenished with new ones, limited insecticides are available. Therefore, the evaluation procedure of the insecticide resistance should be inexpensive to establish in countries with low resources successfully. The mathematical approach revealed in this study requires only initial monitoring of the *kdr* mutant alleles for the evaluation of phenotypic resistance to pyrethroid insecticides, reducing the cost of the resources. In addition, it further highlights the difficulty of managing the resistance development in the mosquito population.

## Conclusions

The current research highlights the need for systematic and routine insecticide resistance surveillance on a finer geographical scale with the aid of the rational approximations created for the specific area.

An initial pilot study is recommended for a certain region of interest to build the best-fitted rational approximation model, which can be used as the reference there onwards. Therefore, the results of random sampling of dengue vector mosquitoes can be used to predict the insecticide resistance of that specific study region. This prediction helps to infer the resistance status despite the robust nature of the factors considered. Thereby, the health authorities will be able to develop many appropriate insecticide application strategies in combination with other control measures. In this study, even though only 10 populations were considered, > 400 mosquito samples were analyzed. In future, the predictions can be further enhanced by increasing the number of populations used.

### Supplementary Information

**Additional file 1: Table S1.** Mean percentages of mortality and standard deviation at 24 h for impregnated paper tests per insecticide, *Aedes aegypti* population, and year. The total number of exposed mosquitoes is indicated (n).

**Additional file 2: Table S2.** Haplotypes present when F1534C and V1016G mutations are concerned in the pyrethroid-resistant *Aedes aegypti* mosquitoes of the three study sites in 2017 and 2020.

**Additional file 3: Table S3.** Haplotypes present when F1534C, V1016G, and S989P mutations are concerned in the pyrethroid-resistant *Aedes aegypti* mosquitoes of the three study site in 2017.

## Data Availability

All data generated or analyzed during this study are included in this published article and its supplementary information files.

## Abbreviations

ASPCR: Allele-specific polymerase chain reaction
DDT: Dichlorodiphenyltrichloroethane
MOH: Medical Officer of Health
OP: Organophosphates
PHI: Public Health Inspector
PY: Pyrethroid
TBE: Tris–borate–EDTA
TE: Tris EDTA
ULV: Ultra-low-volume spraying
WHO: World Health Organization
