# Supplementary material for: Fine-scale monitoring of insecticide resistance in Aedes aegypti (Diptera: Culicidae) from Sri Lanka and modeling the phenotypic resistance using rational approximation
Source: Parasit Vectors. 2024 Jan 12;17:18. doi: 10.1186/s13071-023-06100-9 (PMC10785423; doi:10.1186/s13071-023-06100-9)
Supplement: Supplementary file 1 — Additional file 1: Table S1. Mean percentages of mortality and standard deviation at 24 h for impregnated paper tests per insecticide, Aedes aegypti population, and year. The total number of exposed mosquitoes is indicated (n). [file 13071_2023_6100_MOESM1_ESM.pdf]

S1 Table. Mean percentages of mortality and standard deviation at 24 h for impregnated paper tests per insecticide, *Aedes aegypti* population and year. The total number of exposed mosquitoes is indicated (n).

Insecticide	Dose	Population	Year	n	Mean 24 h M (%) + SD
**permethrin**	0.75%	Delkada	2017	100	28.03 ± 0.003
		Gangodawila	2017	100	51.45 ± 0.01
		Udahamulla	2017	100	54.35 ± 1.01
	0.25%	Delkada	2020	100	08 ± 1.00
		Gangodawila	2020	100	25 ± 2.71
		Udahamulla	2020	100	00 ± 0.00
	1.25%(5x)	Delkada	2020	100	17 ± 3.30
		Gangodawila	2020	100	49 ± 1.71
		Udahamulla	2020	100	12.05 ± 1.91
**deltamethrin**	0.05%	Delkada	2017	100	84.68 ± 0.01
		Gangodawila	2017	100	66.32 ± 0.01
		Udahamulla	2017	100	59.19 ± 0.03
	0.03%	Delkada	2020	100	40 ± 2.58
		Gangodawila	2020	100	45 ± 3.37
		Udahamulla	2020	100	41 ± 5.50
	0.15%(5x)	Delkada	2020	100	97 ± 1.50
		Gangodawila	2020	100	100 ± 0.00
		Udahamulla	2020	100	85 ± 2.00
**malathion**	5%	Delkada	2017	100	100 ± 0.00
		Gangodawila	2017	100	100 ± 0.00
		Udahamulla	2017	100	98.77 ± 0.003
	0.8%	Delkada	2020	100	77 ± 1.893
		Gangodawila	2020	100	31 ± 4.990
		Udahamulla	2020	100	66 ± 1.893
