# Supplementary material for: Fine-scale monitoring of insecticide resistance in Aedes aegypti (Diptera: Culicidae) from Sri Lanka and modeling the phenotypic resistance using rational approximation
Source: Parasit Vectors. 2024 Jan 12;17:18. doi: 10.1186/s13071-023-06100-9 (PMC10785423; doi:10.1186/s13071-023-06100-9)
Supplement: Supplementary file 2 — Additional file 2: Table S2. Haplotypes present when F1534C and V1016G mutations are concerned in the pyrethroid-resistant Aedes aegypti mosquitoes of the three study sites in 2017 and 2020. [file 13071_2023_6100_MOESM2_ESM.pdf]

S2 Table. Haplotypes present when F1534C and V1016G mutations are concerned in the pyrethroid-resistant *Ae. aegypti* mosquitoes of the three study site in the year 2017 and 2020.

Phenotype		Year	Delkanda Resistant	Gangodawila Resistant	Udahamulla Resistant
No of mosquitoes		2017	34	50	30
		2020	24	46	46
FF/GG	No	2017	1	1	1
		2020	0	0	1
	Frequency	2017	0.0294	0.0200	0.0333
		2020	0.0000	0.0000	0.0217
FF/VV	No	2017	0	0	0
		2020	1	1	1
	Frequency	2017	0.0000	0.0000	0.0000
		2020	0.0417	0.0217	0.0217
FF/VG	No	2017	0	1	0
		2020	2	1	4
	Frequency	2017	0.0000	0.0200	0.0000
		2020	0.0833	0.0217	0.0870
FC/VV	No	2017	4	15	1
		2020	2	7	3
	Frequency	2017	0.1176	0.3000	0.0333
		2020	0.0833	0.1522	0.0652
FC/VG	No	2017	9	9	11
		2020	0	2	18
	Frequency	2017	0.2647	0.1800	0.3667
		2020	0.0000	0.0435	0.3913
FC/GG	No	2017	1	2	0
		2020	0	3	3
	Frequency	2017	0.0294	0.0400	0.0000
		2020	0.0000	0.0652	0.0652
CC/VV	No	2017	14	12	8
		2020	11	21	4
	Frequency	2017	0.4118	0.2400	0.2667
		2020	0.4583	0.4565	0.0870
CC/VG	No	2017	5	9	8
		2020	3	6	7
	Frequency	2017	0.1471	0.1800	0.2667
		2020	0.1250	0.1304	0.1522
CC/GG	No	2017	0	1	1
		2020	5	5	5
	Frequency	2017	0.0000	0.0200	0.0333
		2020	0.2083	0.1087	0.1087
