# Supplementary material for: Fine-scale monitoring of insecticide resistance in Aedes aegypti (Diptera: Culicidae) from Sri Lanka and modeling the phenotypic resistance using rational approximation
Source: Parasit Vectors. 2024 Jan 12;17:18. doi: 10.1186/s13071-023-06100-9 (PMC10785423; doi:10.1186/s13071-023-06100-9)
Supplement: Supplementary file 3 — Additional file 3: Table S3. Haplotypes present when F1534C, V1016G, and S989P mutations are concerned in the pyrethroid-resistant Aedes aegypti mosquitoes of the three study site in 2017. [file 13071_2023_6100_MOESM3_ESM.pdf]

**S3 Table. Haplotypes present when F1534C, V1016G and S989P mutations are concerned in the pyrethroid-resistant *Ae. aegypti* mosquitoes of the three study site in the year 2017.**

| Phenotype | | Delkanda Resistant | Gangodawila Resistant | Udahamulla Resistant |
|---|---|---|---|---|
| No of mosquitoes | | 17 | 43 | 20 |
| FF/GG/PP | No | 1 | 2 | 1 |
| | Frequency | 0.06 | 0.05 | 0.05 |
| FF/VV/SS | No | 0 | 1 | 0 |
| | Frequency | 0.00 | 0.02 | 0.00 |
| FF/VG/SS | No | 0 | 1 | 0 |
| | Frequency | 0.00 | 0.02 | 0.00 |
| FF/GG/SS | No | 0 | 0 | 0 |
| | Frequency | 0.00 | 0.00 | 0.00 |
| FC/VV/SS | No | 3 | 7 | 1 |
| | Frequency | 0.18 | 0.16 | 0.05 |
| FC/VV/SP | No | 0 | 0 | 0 |
| | Frequency | 0.00 | 0.00 | 0.00 |
| FC/VG/SS | No | 2 | 4 | 4 |
| | Frequency | 0.12 | 0.09 | 0.20 |
| FC/VG/SP | No | 0 | 2 | 1 |
| | Frequency | 0.00 | 0.05 | 0.05 |
| FC/GG/SP | No | 1 | 0 | 0 |
| | Frequency | 0.06 | 0.00 | 0.00 |
| FC/GG/SS | No | 0 | 1 | 0 |
| | Frequency | 0.00 | 0.02 | 0.00 |
| FC/GG/PP | No | 0 | 1 | 0 |
| | Frequency | 0.00 | 0.02 | 0.00 |
| CC/VV/SS | No | 10 | 18 | 8 |
| | Frequency | 0.59 | 0.42 | 0.40 |
| CC/VV/SP | No | 0 | 0 | 0 |
| | Frequency | 0.00 | 0.00 | 0.00 |
| CC/VG/SS | No | 0 | 5 | 5 |
| | Frequency | 0.00 | 0.12 | 0.25 |
| CC/VG/SP | No | 0 | 1 | 0 |
| | Frequency | 0.00 | 0.02 | 0.00 |
| CC/GG/SS | No | 0 | 0 | 0 |
| | Frequency | 0.00 | 0.00 | 0.00 |
